# Supplementary material for: Using i-GONAD for Cell-Type-Specific and Systematic Analysis of Developmental Transcription Factors In Vivo
Source: Biology (Basel). 2023 Sep 13;12(9):1236. doi: 10.3390/biology12091236 (PMC10526018; doi:10.3390/biology12091236)
Supplement: Supplementary file 1 [file biology-12-01236-s001.zip › biology-2582423-supplementary.pdf]

Supplementary Materials

Using *i*-GONAD for Cell Type-Specific and Systematic Analysis of Developmental Transcription Factors *in Vivo*

Christoph Wiegreffe, Simon Ehricke, Luisa Schmid, Jacqueline Andratschke and Stefan Britsch

Figure S1
Figure S2
Figure S3
Figure S4
Figure S5
Figure S6
Figure S7
Figure S8
Figure S9
Figure S10
Figure S11
Figure S12
Figure S13
Figure S14
Figure S15
Figure S16
Figure S17

Table S1
Table S2
Table S3
Table S4

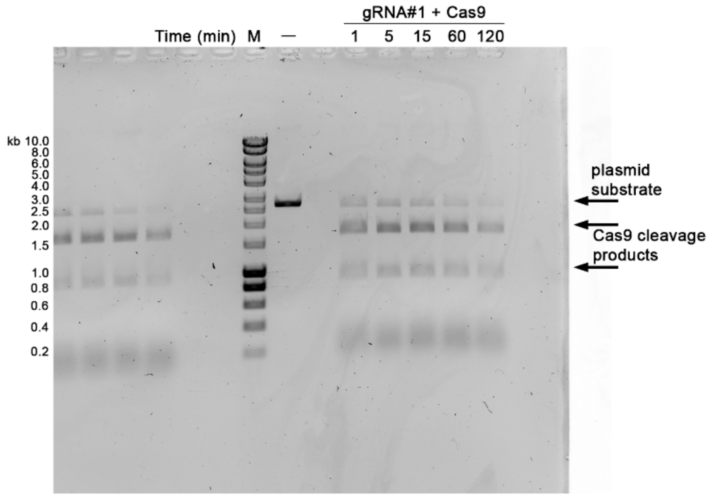

Figure S1. Full DNA gel image of endonuclease activity assay of Cas9 and gRNA#1 (related to Figure 1C). Samples were taken at indicated time points and resolved in a 1% agarose gel. M, DNA size marker.

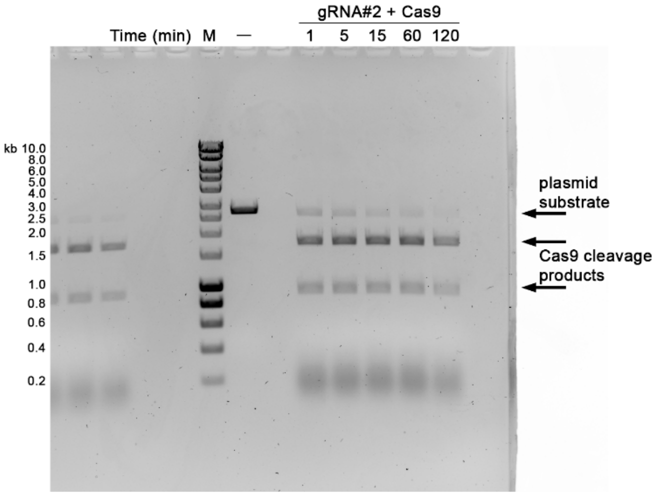

Figure S2. Full DNA gel image of endonuclease activity assay of Cas9 and gRNA#2 (related to Figure 1E). Samples were taken at indicated time points and resolved in a 1% agarose gel. M, DNA size marker.

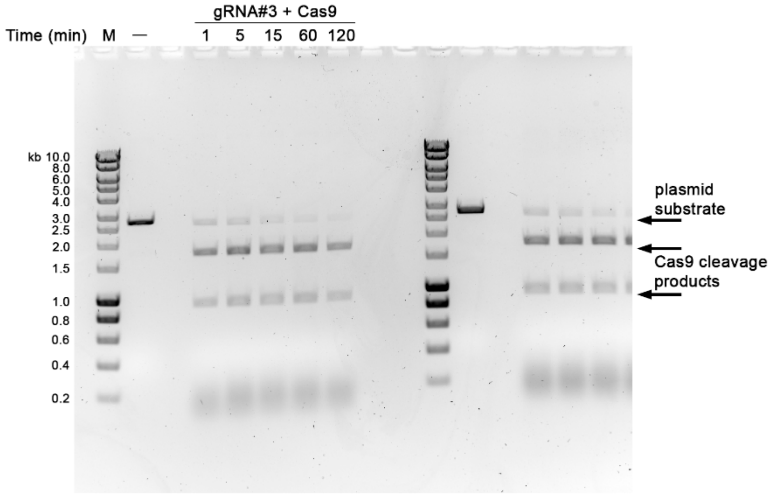

Figure S3. Full DNA gel image of endonuclease activity assay of Cas9 and gRNA#3 (related to Figure 1E). Samples were taken at indicated time points and resolved in a 1% agarose gel. M, DNA size marker.

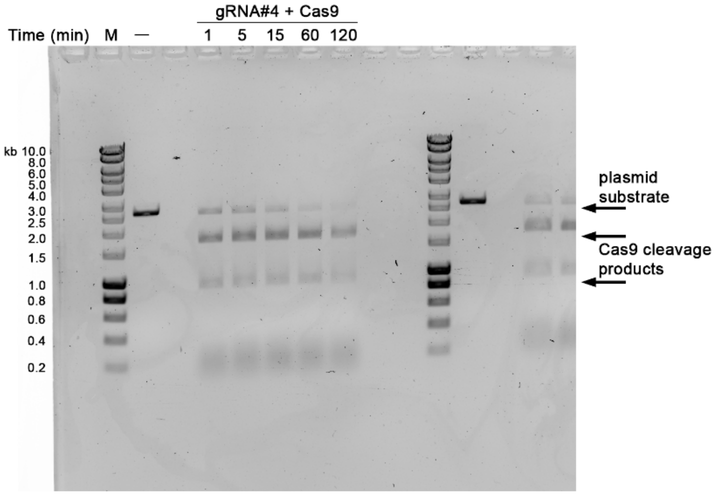

Figure S4. Full DNA gel image of endonuclease activity assay of Cas9 and gRNA#4 (related to Figure 1E). Samples were taken at indicated time points and resolved in a 1% agarose gel. M, DNA size marker.

A

	Bcl11b			
	gRNA#5	gRNA#6	gRNA#7	gRNA#8
specificity score	98.40%	100%	96.67%	99.60%
activity score	0.445	0.341	0.642	0.491
distance to insertion site	7	10	11	12

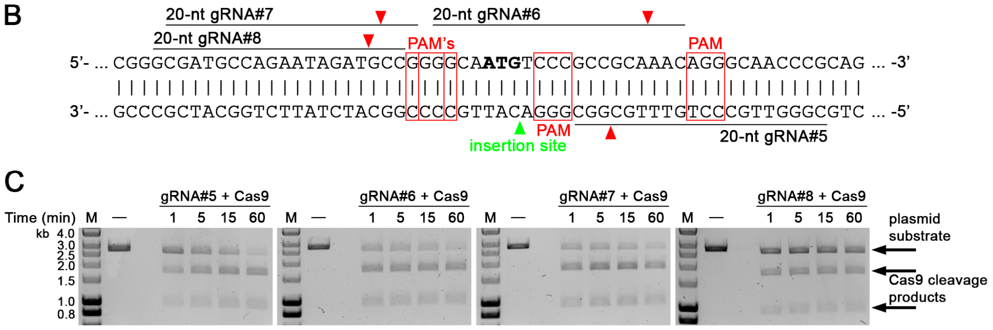

Figure S5. Validation of candidate gRNAs targeting the 5′ end of the coding region of Bcl11b. (**A**) In silico identified candidate gRNAs targeting the 5′ end of the protein coding region of Bcl11b. (**B**) Sequence of the target sites of gRNA#5, -#6, -#7, and -#8 in plasmid substrate. Cas9 cleavage and intended insertion sites are indicated by red and green arrowheads, respectively. PAM motifs are highlighted with red boxes and start codon shown in bold. (**C**) Endonuclease activity assay of Cas9 using ScaI-linearized plasmid (2693 bp). Samples were taken at indicated time points. Cas9 cleavage products (gRNA#5: 1750 and 943 bp; gRNA#6: 1747 and 946 bp; gRNA#7: 1768 and 925 bp; gRNA#8: 1769 and 924 bp) were resolved in a 1% agarose gel. M, DNA size marker.

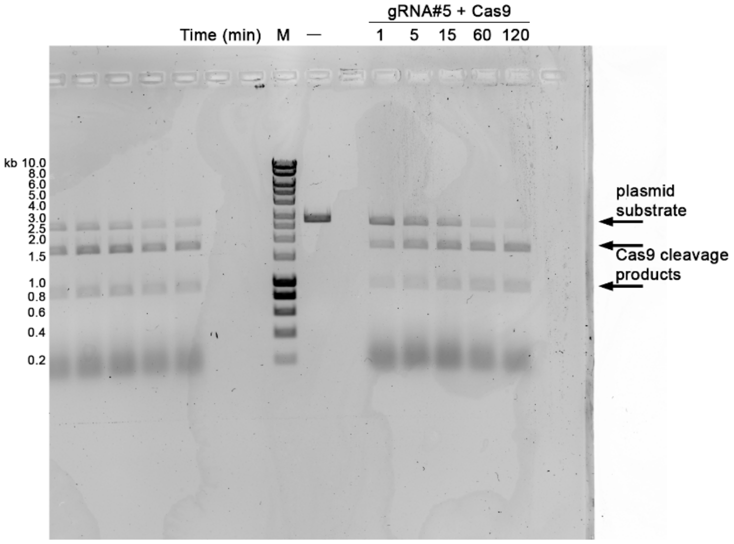

Figure S6. Full DNA gel image of endonuclease activity assay of Cas9 and gRNA#5 (related to Supplementary Materials, Figure S5C). Samples were taken at indicated time points and resolved in a 1% agarose gel. M, DNA size marker.

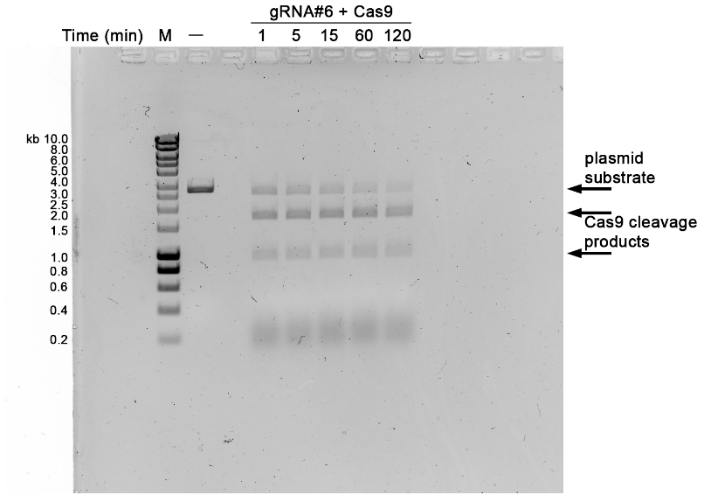

Figure S7. Full DNA gel image of endonuclease activity assay of Cas9 and gRNA#6 (related to Supplementary Materials, Figure S5C). Samples were taken at indicated time points and resolved in a 1% agarose gel. M, DNA size marker.

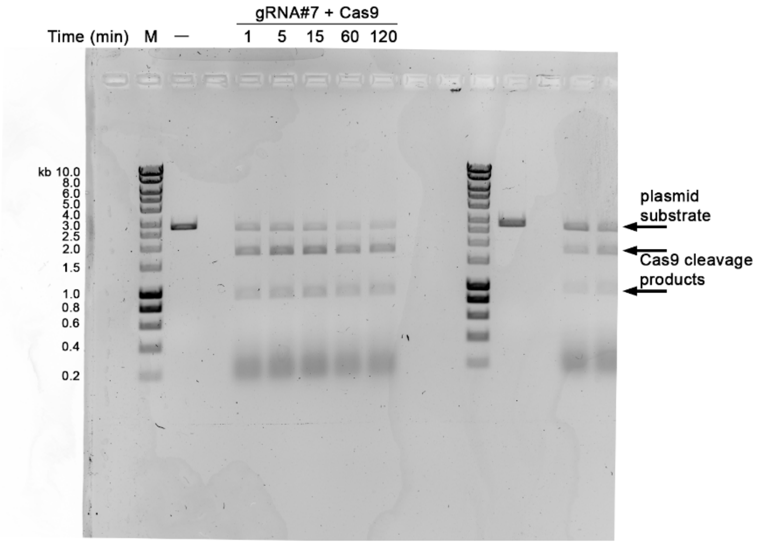

Figure S8. Full DNA gel image of endonuclease activity assay of Cas9 and gRNA#7 (related to Supplementary Materials, Figure S5C). Samples were taken at indicated time points and resolved in a 1% agarose gel. M, DNA size marker.

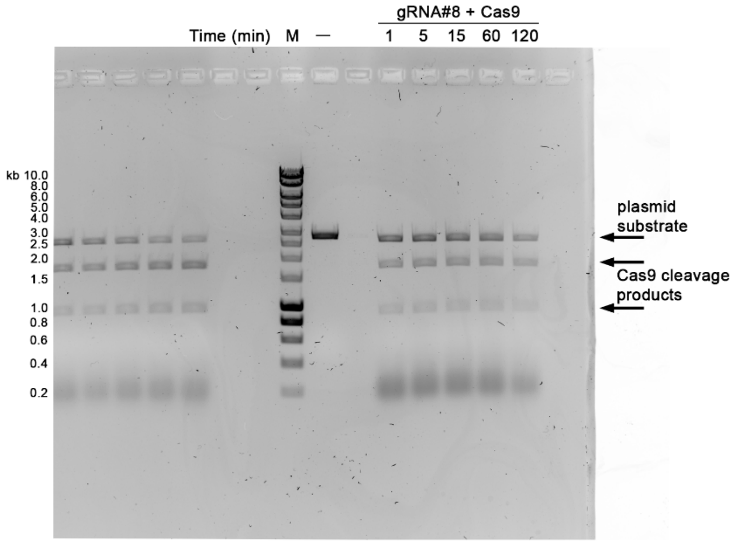

Figure S9. Full DNA gel image of endonuclease activity assay of Cas9 and gRNA#8 (related to Supplementary Materials, Figure S5C). Samples were taken at indicated time points and resolved in a 1% agarose gel. M, DNA size marker.

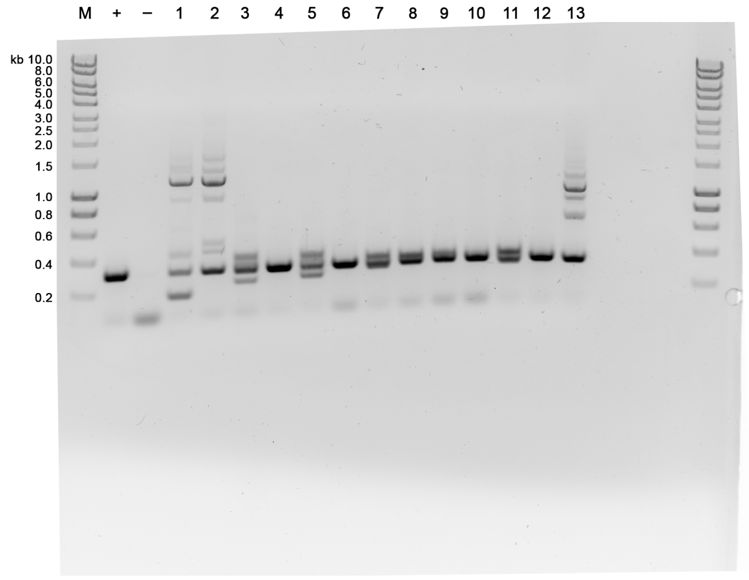

Figure S10. Full DNA gel image of representative genotyping analysis of F0 generation with Bcl11a$^{T2A\text{-}EGFPnuc}$ allele (related to Figure 3C). Samples were taken at indicated time points and resolved in a 1% agarose gel. Expected fragment size of knock-in allele: 1126 bp. +, positive control (301 bp); -, negative control; M, size marker.

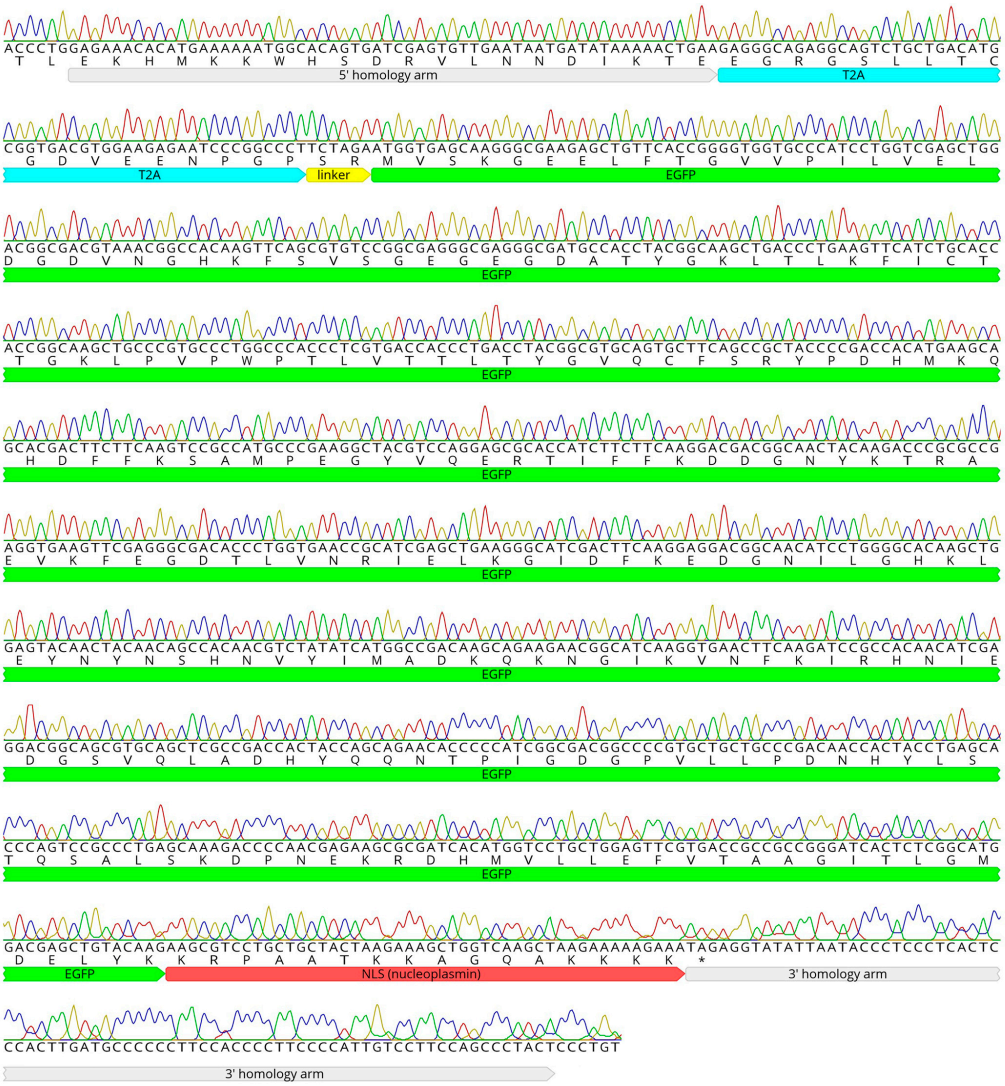

Figure S11. Sequencing chromatogram of Bcl11a$^{T2A\text{-}EGFPnuc}$ allele (related to Figure 3). Representative sequencing chromatogram showing 5′ and 3′ junctional regions of the T2A-EGFPnuc cassette inserted into the Bcl11a locus.

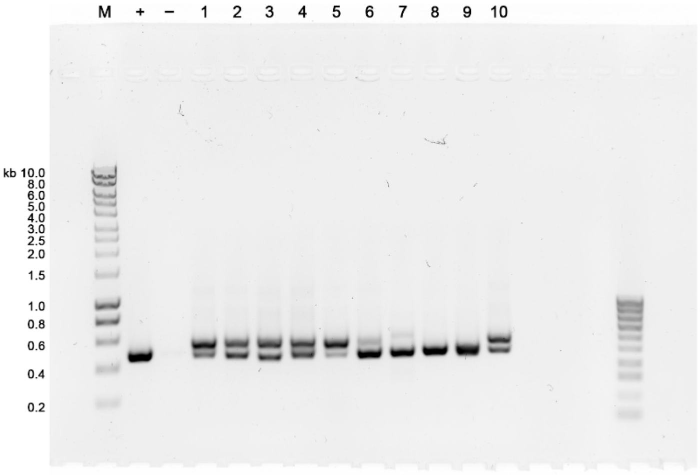

Figure S12. Full DNA gel image of representative genotyping analysis of F0 generation with Bcl11a^{FLBIO} allele (related to Figure 4C). Samples were taken at indicated time points and resolved in a 1% agarose gel. Expected fragment size of knock-in allele: 547 bp. +, positive control (472 bp); -, negative control; M, size marker.

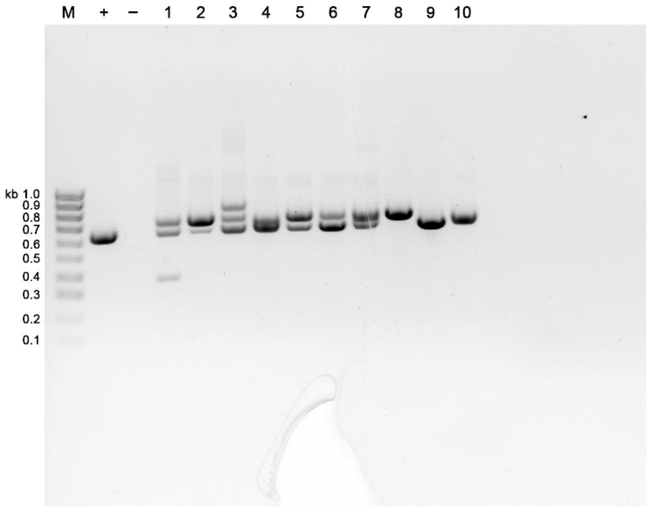

Figure S13. Full DNA gel image of representative genotyping analysis of F0 generation with Bcl11b^{FLBIO} allele (related to Figure 4G). Samples were taken at indicated time points and resolved in a 1% agarose gel. Expected fragment size of knock-in allele: 694 bp. +, positive control (619 bp); -, negative control; M, size marker.

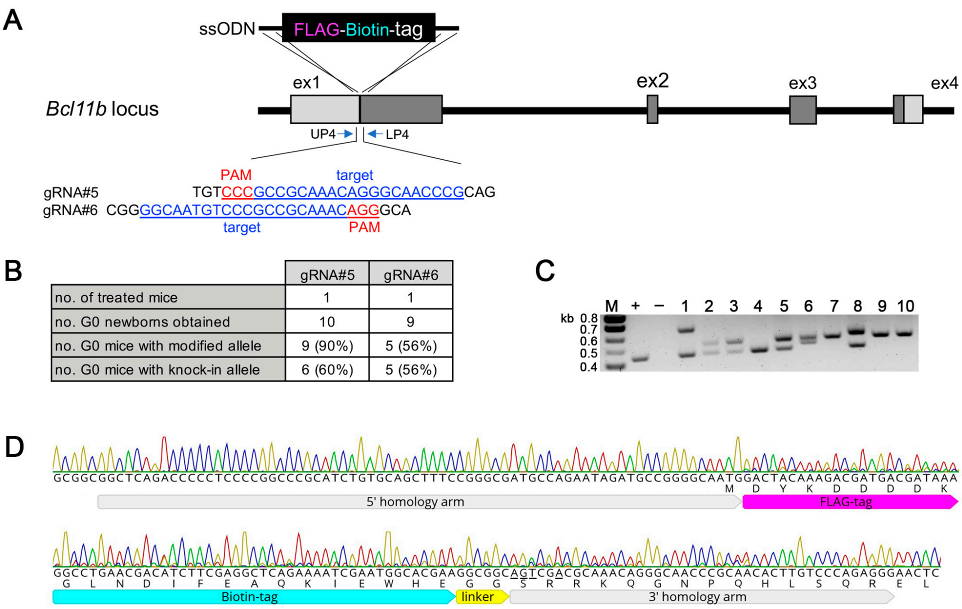

	gRNA#5	gRNA#6
no. of treated mice	1	1
no. G0 newborns obtained	10	9
no. G0 mice with modified allele	9 (90%)	5 (56%)
no. G0 mice with knock-in allele	6 (60%)	5 (56%)

Figure S14. Generation of Bcl11b$^{FLBIO/+}$ mice expressed N-terminally FLBIO-tagged Bcl11b. (**A**) Targeting scheme showing insertion of a FLAG-Biotin-tag at the 5′ end of the coding region of the Bcl11b locus. The target sequence of gRNA#5, gRNA#6, and the location of the genotyping primers are shown. A ssODN was used as the donor DNA. (**B**) Genome editing efficiency of the 5′ Bcl11b locus by the *i*-GONAD method. (**C**) Representative genotyping analysis of F0 generation (generated with gRNA#5). Expected fragment size of knock-in allele: 498 bp. +, positive control (423 bp); -, negative control; M, DNA size marker. (**D**) Representative sequencing chromatogram showing 5′ and 3′ junctional regions of the inserted FLAG-Biotin-tag from F0-#3 in (**E**) is shown. Silent mutations introduced to avoid internal homology between the Cas9 cleavage and intended insertion sites are underlined.

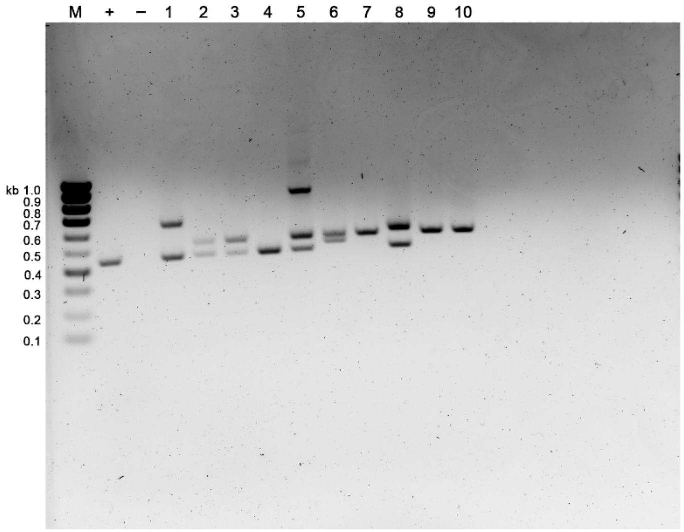

Figure S15. Full DNA gel image of representative genotyping analysis of F0 generation with N-terminally FLBIO-tagged Bcl11b (related to Supplementary Materials, Figure S14C). Samples were taken at indicated time points and resolved in a 1% agarose gel. Expected fragment size of knock-in allele: 498 bp. +, positive control (423 bp); -, negative control; M, size marker.

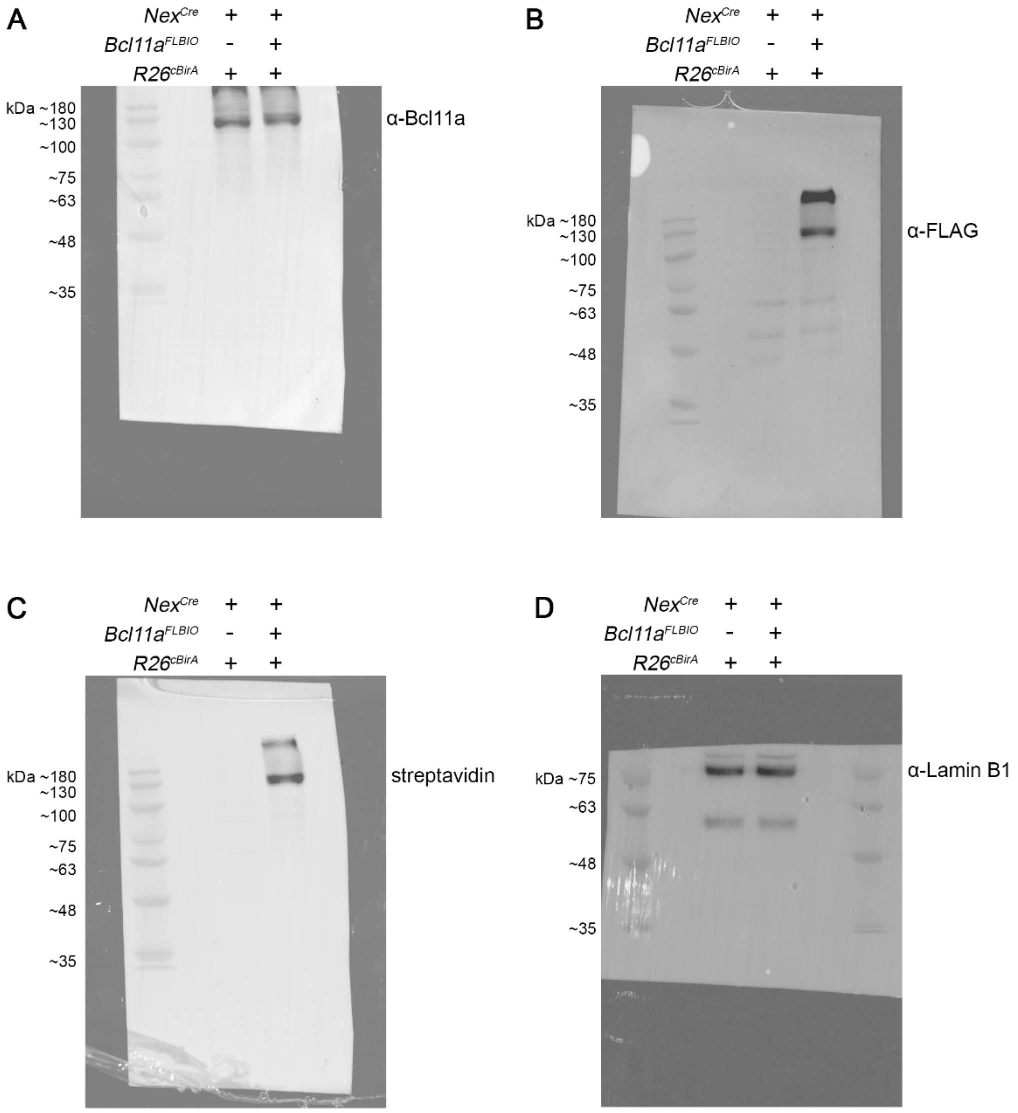

Figure S16. Full Western blot images of biotinylated FLBIO-tagged Bcl11a (related to Figure 5C). Protein lysates from $Nex^{Cre/+}$; $Bcl11a^{+/+}$; $R26^{cBirA/+}$ and $Nex^{Cre/+}$; $Bcl11a^{FLBIO/+}$; $R26^{cBirA/+}$ neocortex were separated by SDS-PAGE, transferred to PVDF membranes (Millipore) by wet electroblotting, and stained with (**A**) mouse anti-Bcl11a antibody (ab18688, Abcam), (**B**) rabbit anti-FLAG antibody (F7425, Sigma), (**C**) streptavidin (Jackson ImmunoResearch), and (**D**) rabbit anti-Lamin B1 antibody (an16048, Abcam) as loading control. Membranes, were incubated with peroxidase-conjugated secondary antibodies (Jackson ImmunoResearch) and Pierce ECL Western Blotting Substrate (Thermo) was used for signal detection. Chemiluminescence and brightfield images were merged to display protein marker (Nippon Genetics).

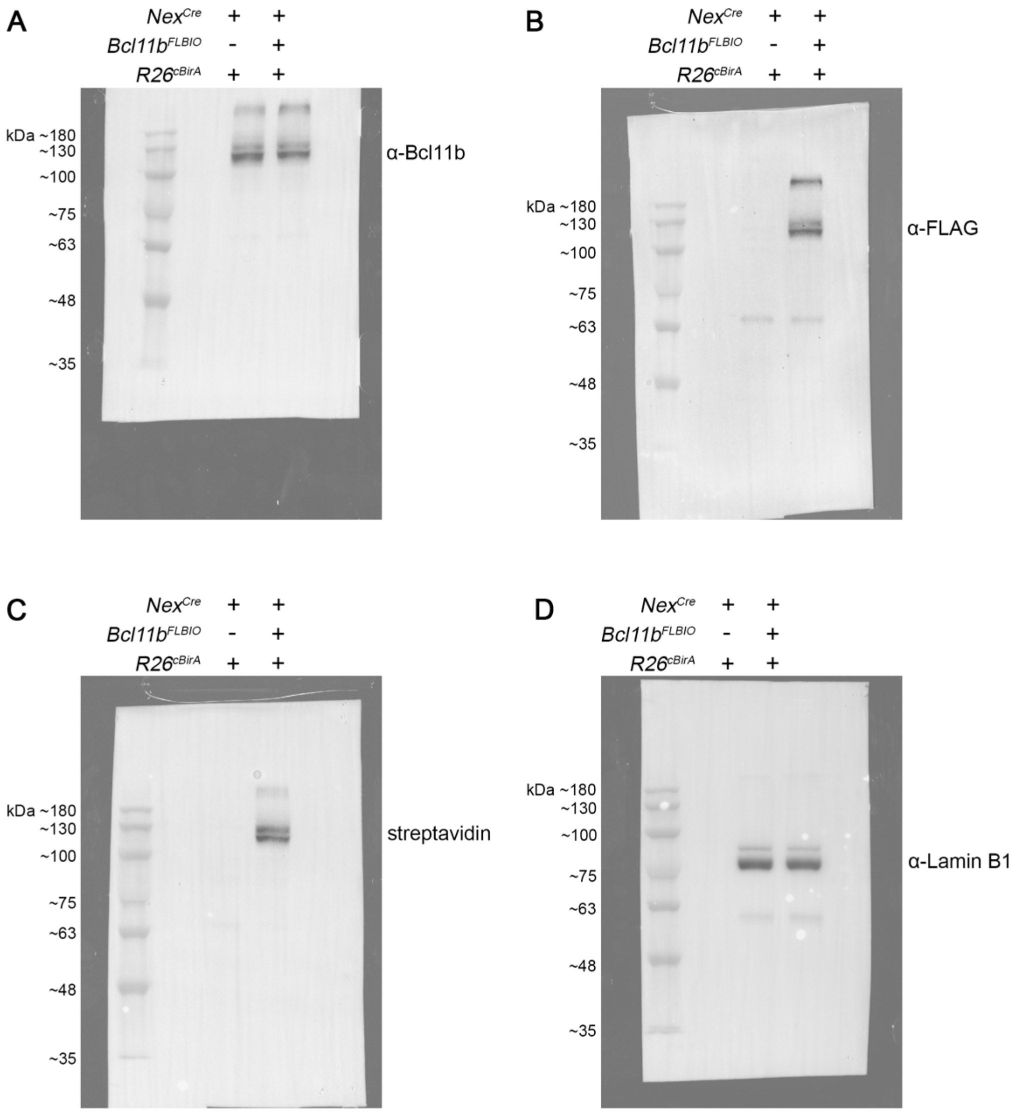

Figure S17. Full Western blot images of biotinylated FLBIO-tagged Bcl11b (related to Figure 5D). Protein lysates from $Nex^{Cre/+}$; $Bcl11b^{+/+}$; $R26^{cBirA/+}$ and $Nex^{Cre/+}$; $Bcl11b^{FLBIO/+}$; $R26^{cBirA/+}$ neocortex were separated by SDS-PAGE, transferred to PVDF membranes (Millipore) by wet electroblotting, and stained with (**A**) rat anti-Bcl11b antibody (ab18465, Abcam), (**B**) rabbit anti-FLAG antibody (F7425, Sigma), (**C**) streptavidin (Jackson ImmunoResearch), and (**D**) rabbit anti-Lamin B1 antibody (an16048, Abcam) as loading control. Membranes, were incubated with peroxidase-conjugated secondary antibodies (Jackson ImmunoResearch) and Pierce ECL Western Blotting Substrate (Thermo) was used for signal detection. Chemiluminescence and brightfield images were merged to display protein marker (Nippon Genetics).

Table S1. Nucleotide sequences of gRNAs.

name	sequence (5' -> 3')	target site	related figures
gRNA#1	ATGATATAAAAACTGAATAG	3' end of Bcl11a protein coding region	1A-C; 3A; S1
gRNA#2	GAGGAGCTAAGCGCATACGT	3' end of Bcl11b protein coding region	1A,D,E; 3E; S2
gRNA#3	AGAGGAGCTAAGCGCATACG	3' end of Bcl11b protein coding region	1A,D,E; 3E; S3
gRNA#4	GGAGCTAAGCGCATACGTGG	3' end of Bcl11b protein coding region	1A,D,E; S4
gRNA#5	CGGGTTGCCCTGTTTGCGGC	5' end of Bcl11b protein coding region	S5A-C; S6; S14A
gRNA#6	GGCAATGTCCCGCCGCAAAC	5' end of Bcl11b protein coding region	S5A-C; S7; S14A
gRNA#7	CGATGCCAGAATAGATGCCG	5' end of Bcl11b protein coding region	S5A-C; S8
gRNA#8	GCGATGCCAGAATAGATGCC	5' end of Bcl11b protein coding region	S5A-C; S9

Table S2. Nucleotide sequences of ssODNs. Insertion sequences are underlined.

name	sequence (5' -> 3')	related figures
Bcl11a$^{3'FLBIO\text{-}gRNA\#1}$	TACAGTACCCTGGAGAAACACATGAAAAAATGGCACAGTGATCGAGTGTTGAATAATGATAT<br>AAAAACTGAAGGCGGCGACTACAAAGACGATGACGATAAAGGCCTGAACGACATCTTCGAGG<br>CTCAGAAAATCGAATGGCACGAATAGAGGTATATTAATACCCTCCCTCACTCCCACTTG	4A,D
Bcl11b$^{3'FLBIO\text{-}gRNA\#2/3/4}$	AAACACATGAAAAAGTGGCACGGTGAACACTTGCTGACTAATGATGTCAAAATCGAGCAGGC<br>TGAGAGGAGCGGCGGCGACTACAAAGACGATGACGATAAAGGCCTGAACGACATCTTCGAGG<br>CTCAGAAAATCGAATGGCACGAATAACGTCTAACGTGGGGGACACTGCGTGCGTGCGTCTGT<br>ACAGCGT	4E,H
Bcl11b$^{5'FLBIO\text{-}gRNA\#5}$	GGCTCAGACCCCCTCCCCGGCCCGCATCTGTGCAGCTTTCCGGGCGATGCCAGAATAGATGC<br>CGGGGCAATGGACTACAAAGACGATGACGATAAAGGCCTGAACGACATCTTCGAGGCTCAGA<br>AAATCGAATGGCACGAAGGCGGCAGTCGACGCAAACAGGGCAACCCGCAGCACTTGTCCCAG<br>AGGGAAC	S14A,D
Bcl11b$^{5'FLBIO\text{-}gRNA\#6}$	GGCTCAGACCCCCTCCCCGGCCCGCATCTGTGCAGCTTTCCGGGCGATGCCAGAATAGATGC<br>CGGGGCAATGGACTACAAAGACGATGACGATAAAGGCCTGAACGACATCTTCGAGGCTCAGA<br>AAATCGAATGGCACGAAGGCGGCTCCAGAAGAAAACAGGGCAACCCGCAGCACTTGTCCCAG<br>AGGGAAC	S14A

Table S3. Nucleotide sequence of plasmid for generating ssDNA. T7 promoter in bold, insertion sequence underlined, plasmid backbone in lower case.

name	sequence (5' -> 3')	related figures
pEX-A128-Bcl11a$^{T2A-EGFPnuc}$	cttaag**TAATACGACTCACTATAG**GAGAAACACATGAAAAAATGGCACAGTGATCGAGTGTTGAA<br>TAATGATATAAAAACTGAAGAGGGCAGAGGCAGTCTGCTGACATGCGGTGACGTGGAAGAGAATC<br>CCGGCCCTTCTAGAATGGTGAGCAAGGGCGAAGAGCTGTTCACCGGGGTGGTGCCCATCCTGGTC<br>GAGCTGGACGGCGACGTAAACGGCCACAAGTTCAGCGTGTCCGGCGAGGGCGAGGGCGATGCCAC<br>CTACGGCAAGCTGACCCTGAAGTTCATCTGCACCACCGGCAAGCTGCCCGTGCCCTGGCCCACCC<br>TCGTGACCACCCTGACCTACGGCGTGCAGTGCTTCAGCCGCTACCCCGACCACATGAAGCAGCAC<br>GACTTCTTCAAGTCCGCCATGCCCGAAGGCTACGTCCAGGAGCGCACCATCTTCTTCAAGGACGA<br>CGGCAACTACAAGACCCGCGCCGAGGTGAAGTTCGAGGGCGACACCCTGGTGAACCGCATCGAGC<br>TGAAGGGCATCGACTTCAAGGAGGACGGCAACATCCTGGGGCACAAGCTGGAGTACAACTACAAC<br>AGCCACAACGTCTATATCATGGCCGACAAGCAGAAGAACGGCATCAAGGTGAACTTCAAGATCCG<br>CCACAACATCGAGGACGGCAGCGTGCAGCTCGCCGACCACTACCAGCAGAACACCCCCATCGGCG<br>ACGGCCCCGTGCTGCTGCCCGACAACCACTACCTGAGCACCCAGTCCGCCCTGAGCAAAGACCCC<br>AACGAGAAGCGCGATCACATGGTCCTGCTGGAGTTCGTGACCGCCGCCGGGATCACTCTCGGCAT<br>GGACGAGCTGTACAAGAAGCGTCCTGCTGCTACTAAGAAAGCTGGTCAAGCTAAGAAAAAGAAAT<br>AGAGGTATATTAATACCCTCCCTCACTCCCACTTGATGCCCCCCTTCCACCCCTTCCCCATTGTC<br>CTTCCAGCCCTACTgctagcacctgcttttgctcgcttggatccgaattcaaaggtgaaattgtt<br>atccgctcacaattccacacaacatacgagccggaagcataaagtgtaaagcctggggtgcctaa<br>tgagtgagctaactcacattaattgcgttgcgctcactgcccgctttccagtcgggaaacctgtc<br>gtgccagctgcattaatgaatcggccaacgcgcggggagaggcggtttgcgtattgggcgctctt<br>ccgcttcctcgctcactgactcgctgcgctcggtcgttcggctgcggcgagcggtatcagctcac<br>tcaaaggcggtaatacggttatccacagaatcaggggataacgcaggaaagaacatgtgagcaaa<br>aggccagcaaaaggccaggaaccgtaaaaaggccgcgttgctggcgtttttccataggctccgcc<br>cccctgacgagcatcacaaaaatcgacgctcaagtcagaggtggcgaaacccgacaggactataa<br>agataccaggcgtttccccctggaagctccctcgtgcgctctcctgttccgaccctgccgcttac<br>cggatacctgtccgcctttctcccttcgggaagcgtggcgctttctcatagctcacgctgtaggt<br>atctcagttcggtgtaggtcgttcgctccaagctgggctgtgtgcacgaaccccccgttcagccc<br>gaccgctgcgccttatccggtaactatcgtcttgagtccaacccggtaagacacgacttatcgcc<br>actggcagcagccactggtaacaggattagcagagcgaggtatgtaggcggtgctacagagttct<br>tgaagtggtggcctaactacggctacactagaagaacagtatttggtatctgcgctctgctgaag<br>ccagttaccttcggaaaaagagttggtagctcttgatccggcaaacaaaccaccgctggtagcgg<br>tggtttttttgtttgcaagcagcagattacgcgcagaaaaaaaggatctcaagaagatcctttga<br>tcttttctacggggtctgacgctcagtggaacgaaaactcacgttaagggattttggtcatgaga<br>ttatcaaaaaggatcttcacctagatccttttaaattaaaaatgaagttttaaatcaatctaaag<br>tatatatgagtaaacttggtctgacagttaccaatgcttaatcagtgaggcacctatctcagcga<br>tctgtctatttcgttcatccatagttgcctgactccccgtcgtgtagataactacgatacgggag<br>ggcttaccatctggccccagtgctgcaatgataccgcgactcccacgctcaccggctccagattt<br>atcagcaataaaccagccagccggaagggccgagcgcagaagtggtcctgcaactttatccgcct<br>ccatccagtctattaattgttgccgggaagctagagtaagtagttcgccagttaatagtttgcgc<br>aacgttgttgccattgctacaggcatcgtggtgtcacgctcgtcgtttggtatggcttcattcag<br>ctccggttcccaacgatcaaggcgagttacatgatcccccatgttgtgcaaaaaagcggttagct<br>ccttcggtcctccgatcgttgtcagaagtaagttggccgcagtgttatcactcatggttatggca<br>gcactgcataattctcttactgtcatgccatccgtaagatgcttttctgtgactggtgagtactc<br>aaccaagtcattctgagaatagtgtatgcggcgaccgagttgctcttgcccggcgtcaatacggg<br>ataataccgcgccacatagcagaactttaaaagtgctcatcattggaaaacgttcttcggggcga<br>aaactctcaaggatcttaccgctgttgagatccagttcgatgtaacccactcgtgcacccaactg<br>atcttcagcatcttttactttcaccagcgtttctgggtgagcaaaaacaggaaggcaaaatgccg<br>caaaaaagggaataagggcgacacggaaatgttgaatactcatactcttcctttttcaatattat<br>tgaagcatttatcagggttattgtctcatgagcggatacatatttgaatgtatttagaaaaataa<br>acaaataggggttccgcgcacatttccccgaaaagtgccacctgacgtctaagaaaccattatta<br>tcatgacattaacctataaaaataggcgtatcacgaggccctttcgtctcgcgcgtttcggtgat<br>gacggtgaaaacctctgacacatgcagctcccggagacggtcacagcttgtctgtaagcggatgc<br>cgggagcagacaagcccgtcagggcgcgtcagcgggtgttggcgggtgtcggggctggcttaact<br>atgcggcatcagagcagattgtactgagagaaaggcaattgggtaccgagctcgcggccgcaagc	3A; S11

Table S4. Nucleotide sequences of genotyping primers.

name	sequence (5' -> 3')	amplicon size		related figures
		edited allele	wild-type allele	
UP1	GTGGGGAAGGACGTTTACAA	1126 bp	301 bp	3A,C; S10
LP1	CTGGTGACAAGCACTCATTCA			
UP2	AAATGCGAGCTGTGCAACTA	547 bp	472 bp	4A,C; S12
LP2	CATGCATTCAAACGGTGAGA			
UP3	AGGACTTCGCAGACACAGGT	694 bp	619 bp	4E,G; S13
LP3	ACATCGTCGGAACATTCCTC			
UP4	GATGAGTTCCCTCTGGGACA	498 bp	423 bp	S14A,C; S15
LP4	GGCCAAGCACTTTGAAGAAG			
